# Mindful2Work: Effects of Combined Physical Exercise, Yoga, and Mindfulness Meditations for Stress Relieve in Employees. A Proof of Concept Study

**DOI:** 10.1007/s12671-016-0593-x

**Published:** 2016-08-23

**Authors:** Esther I. de Bruin, Anne R. Formsma, Gerard Frijstein, Susan M. Bögels

**Affiliations:** 1 Research Institute of Child Development and Education (RICDE), Research Priority Area Yield, University of Amsterdam, Nieuwe Achtergracht 127, 1018 WS Amsterdam, The Netherlands; 2 UvA Minds, Academic Outpatient Child And Adolescent Treatment Center of the University of Amsterdam, Plantage Muidergracht 14, 1018 TV Amsterdam, The Netherlands; 3 Occupational Health and Safety Department Academic Medical Center—University of Amsterdam, Plantage Muidergracht 14, 1018 TV Amsterdam, The Netherlands

**Keywords:** Mindfulness, Physical Exercise, Burnout, Work Stress

## Abstract

Work-related stress and associated illness and burnout is rising in western society, with now as much as almost a quarter of European and half of USA’s employees estimated to be at the point of burnout. Mindfulness meditation, yoga, and physical exercise have all shown beneficial effects for work-related stress and illness. This proof of concept study assessed the feasibility, acceptability, and preliminary effects of the newly developed Mindful2Work training, a combination of physical exercise, restorative yoga, and mindfulness meditations, delivered in six weekly group sessions plus a follow-up session. Participants (*n* = 26, four males), referred by company doctors with (work-related) stress and burnout complaints, completed measurements pre and post the intervention, as well as at 6-week (FU1) and 6-month (FU2) follow-up. Results showed very high feasibility and acceptability of the Mindful2Work training. The training and trainers were rated with an 8.1 and 8.4 on a 1–10 scale, respectively, and training dropout rate was zero. Significant improvements with (very) large effect sizes were demonstrated for the primary outcome measures of physical and mental workability, and for anxiety, depression, stress, sleep quality, positive and negative affect, which remained (very) large and mostly increased further over time. Risk for long-term dropout from work (checklist individual strength [CIS]) was 92 % at pre-test, reduced to 67 % at post-test, to 44 % at FU1, and 35 % at FU2, whereas employees worked (RTWI) 65 % of their contract hours per week at pre-test, which increased to 73 % at post-test, 81 % at FU1 and 93 % at FU2. Intensity of home practice or number of attended sessions were not related to training effects. To conclude, the newly developed Mindful2Work training seems very feasible, and acceptable, and although no control group was included, the large effects of Mindful2Work are highly promising.

## Introduction

Feeling tensed, restless, rushed, or overwhelmed as a result of daily stress is very common in western society. The lifestyle in the contemporary 24-h economy is characterized by speed, time pressure, competition, job insecurity, being constantly available due to modern telecommunication, an overload of stimuli, and multi-tasking in different roles that we fulfill (Stansfeld and Candy 2006). Work-related pressure is indicated as the main source of stress in the USA (Aikens et al. 2014). According to the American Institute of Stress, 75 to 90 % of the GP visits in the USA are related to stress (Rosch 2001). The International Labor Organization estimated that 30 % of all work-related disorders are due to stress (Mino et al. 2006). In line, 22 % of the working population in the European Union experience work-related stress in a way that it has a large negative impact on their well-being (European Agency for Safety and Health at Work 2014). These numbers are expected to only go up in the future (Shanafelt et al. 2015).

Stress is a real health hazard. On the short term, stress can lead to complaints such as headaches, flu-like symptoms, muscle tension and strains, increased heart rate and blood pressure (Gura 2002; Schneiderman et al. 2005), sleep problems (Sadeh et al. 2004), or being mentally unstable and irritable (Hassmén et al. 2000). On the long term, stress can lead to severe fatigue and burnout (Leone et al. 2011; Wessely et al. 1998), anxiety and depression (Hammen 2004; Netterstrøm et al. 2008), problems with cognitive and executive functioning (Mcewen and Sapolsky 1995), relationships and family problems (Coyl et al. 2002), somatic complaints like a weakened immune system (Cohen et al. 1991), cardiovascular disorders, digestive problems (Schneiderman et al. 2005), and chronic illnesses (Wolever et al. 2012). Consequences of stress at the workplace can be a loss of productivity, absenteeism, accidents, poorer judgments, errors, interpersonal problems and conflicts, chronic somatic diseases, abuse of alcohol and drugs, and mental diseases (European Agency for Safety and Health at Work 2014; Kalia 2002). The costs of stress are enormous on both a personal and a societal level. Total annual costs of disorders that were caused by stress were estimated to be $660 billion in the USA and €920 billion in Europe (Mino et al. 2006).

Due to the severe consequences and very high costs of work-related stress, as well as its high occurrence, there is an urgent need for effective solutions. Treatment as usual for work-related stress complaints are either person-directed (cognitive behavioral therapy, psychotherapy, counselling, skill training, communication training, social support, and relaxation exercises), organization-directed (work process restructuring, work performance appraisals, work shift readjustments, and job evaluation), or a combination of both (Awa et al. 2010). A meta-analysis by Richardson and Rothstein (2008) on work-related stress management interventions, which included 63 experimental studies, showed that the most popular interventions were the ones with relaxation and meditation techniques (average duration of interventions was 6.5 weeks, with weekly 1–2-h sessions, with a mean effect size of 0.50), though cognitive behavioral interventions appeared to be the most effective (average duration of interventions was 7.5 weeks, with weekly 1–2-h sessions, with an average effect size of 1.16). The popularity of relaxation and meditation techniques is probably because they are easily accessible, easy to implement, and least expensive (Richardson and Rothstein 2008). This is in line with Henriques et al. (2011) who state that interventions that are easily applicable, inexpensive, can be used by a large number of people, and have minimal side effects are preferable. Methods that meet these criteria and have been proven to be effective in reducing stress and its related symptoms (e.g., depression, stress, anxiety, and somatic complaints) are mindfulness, yoga, and physical exercise.

Mindfulness is an intervention that rapidly gained popularity in the last decades in the USA and Europe. Mindfulness has its origin in the 2500-year-old Buddhist tradition. The definition of mindfulness is ‘awareness that arises through deliberately paying attention in the present moment, non-judgmentally’ (Kabat-Zinn 2003). All human beings have the capacity to be fully aware, though the periods that we are fully present are mostly short and sustaining awareness is a special skill (Siegel et al. 2009). Mindful awareness involves a non-judgmental attitude. We tend to judge experiences immediately: we find things pleasant or unpleasant, good or bad. This judging or labeling colors our experience, and as a result, we do not see clearly how things really are. This awareness and attitude are cultivated by formal practices (for instance, sitting meditation, body scan) and informal practices that integrate these practices in daily life (for instance, doing a routine activity mindfully or eating a meal with awareness). During these practices, attention is trained (monitoring, directing, and sustaining) and self-investigation takes place. Due to observing the content of the mind and our inner reactions, we can relate differently to internal events (Fjorback et al. 2011). We do not have much control over our life events and inner turmoil, but we do have control over how we relate to it. Mindfulness will not eliminate life’s pressures, but it can help us respond to them in a more deliberate and calm manner that benefits our mind and body, as well as our relationship with others.

Mindfulness has shown to be effective in the treatment of stress and rumination (Chiesa and Serretti 2009; Delgado et al. 2010), anxiety and depression (Brown and Ryan 2003; Chiesa and Serretti 2011; Hofmann et al. 2010), chronic pain (Kabat-Zinn et al. 1985), enhances immune functioning (Davidson et al. 2003), cognitive functioning (Zeidan et al. 2010), and improves self-compassion (Chiesa and Serretti 2009) and overall mental well-being (Carmody and Baer 2008).

Yoga has its roots in India and is practiced since thousands of years, but it is only since this century that yoga has become very popular in the USA and Europe (Li and Goldsmith 2012). The word yoga (Sanskrit) means ‘unity’ or ‘to unite,’ which refers to the combination of physical postures (Asanas) and breathing techniques that are being executed with full attention. Multiple studies showed that yoga helps to decrease the effects of stress by reducing the level of the stress hormone cortisol (Granath et al. 2006; West et al. 2004), promoting relaxation and sleep (Khalsa 2004), diminishing muscle tension and counteract musculoskeletal disorders (such as repetitive strain injury [RSI]/complaints arm neck shoulder [CANS]) (Gura 2002), boosts immune functioning (Ross and Thomas 2010), controls blood pressure, heart and metabolic rate, improves strength and physical flexibility, and eases somatic complaints (Raub 2002).

Physical exercise has also been shown to effectively reduce stress and its related symptoms. Regular physical exercise decreases symptoms of anxiety and depression (Conn 2010a; Conn 2010b; McDonald and Hodgdon 1991), as well as psychological stress and anger (Hassmén et al. 2000); counters an over reactive stress-response system; and reduces rumination (Mothes et al. 2014). Physical exercise gives energy and at the same time promotes relaxation and better sleep (DiLorenzo et al. 1999; Youngstedt et al. 1997), boosts the immune system (LaPerriere et al. 1990), and enhances cognitive and executive functioning as well as positive affect (Reed and Buck 2009). Regular exercise further enhances cardiovascular and muscular strength (Pober et al. 2004). A meta-analysis by Conn et al. (2009) showed that physical exercise is an effective tool in preventing and reducing work-related stress, as well as reducing the duration of absenteeism from work (Van den Heuvel et al. 2003).

Knowing that stress-related complaints express themselves mentally and physically, an intervention that targets stress on both levels would be expected to be effective. The aim of the current proof of concept study is to examine the effects of a newly developed 6-week training program in which physical exercise, yoga, and mindfulness meditations are combined. Effects on workability as the primary outcome measure are assessed, as well as effects on secondary outcomes of anxiety, depression, stress, sleep, and positive and negative affect in a sample of employees with (work-related) burnout complaints. Workability is defined as (work-related) stress symptoms such as fatigue, lack of concentration, inactivity, lack of motivation, mental as well as physical workability, and the return to work index. Correlations between intensity of home practice, number of attended sessions, and changes in primary outcome measures are also assessed. In addition to these quantitative measures, feasibility (intervention participation) and acceptability (intervention satisfaction) of the Mindful2Work training are examined.

## Method

### Participants

The 26 participants (22 females) in this study were either self-selected (*n* = 6) or referred by their company doctor (*n* = 20), all because of (work-related) stress complaints. Mean age was 44.9 years (*SD* = 10.59, range 26–60). School levels of the participants were the following: 8 % (*n* = 2) pre-university education, 8 % (*n* = 2) intermediate vocational education, 15 % (*n* = 4) higher vocational education, 65 % (*n* = 17) university, and 4 % (*n* = 1) did not report about their educational background. Of all participants, 38 % (*n* = 10) reported chronic physical complaints (i.e., asthma, migraine) and 38 % (*n* = 10) reported (symptoms of) mental illnesses (i.e., depression, burnout, anxiety, personality disorder).

The majority of participants (70.8 %) indicated they exercised already before the training (i.e., going to the gym, running, cycling, or swimming), of which 23.5 % exercised daily and 76.5 % indicated to exercise weekly. As for yoga, 16 % of the participants indicated to practice weekly yoga, and 12 % of the participants indicated to meditate daily before the training started.

### Procedure

Before the first training session, an intake took place, in which the content of the training was explained and motivation for daily home practice was verified. During intake, exclusion criteria were verified. People suffering from acute psychosis, suicidal ideation, current substance abuse, or diagnosed borderline personality disorder were not eligible to take part in this training. This study was approved by the ethical committee of the University of Amsterdam (number 2014-CDE-3250). Measurements were administered online around 1 week before training (pre-test), directly after training (post-test), 6 weeks after training (follow-up 1), and 6 months after the start of the Mindful2Work training (follow-up 2). At follow-up 2, for feasibility reasons, only four out of the total of seven measurements were administered.

#### Intervention

Mindful2Work is a newly developed structured group training program (Formsma et al. 2015) that consists of six weekly sessions of 2 h and a follow-up session, which all consist of three elements: physical exercise (20 min), yoga (20 min), and mindfulness meditation including psycho-education (80 min). The training sessions are mainly scheduled in the morning between 9 and 11 a.m., which is the participants’ time of preference, considering their energy levels are highest in the morning. Besides attending the sessions, participants are asked to practice daily at home. Home practices consist of daily mindfulness practices (about 20 min per day) related to the theme of the session, covering both formal meditations (e.g., sitting with the breath) and informal mindfulness practices (e.g., doing a routine activity mindfully). Additionally, participants are asked to do yoga (10 min) and physical exercise (20 min) at home. The frequency for yoga and physical exercise builds up from once a week during the first half of the training to twice a week during the second half.

The physical exercise component of the Mindful2Work training is based on aerobic exercises that are easily executable and accessible, low-risk regarding to injuries, target all the muscle groups in the body (strength exercises), and improve condition (cardio). The exercises are executed in a park outdoors, outside of the treatment center. The natural surroundings and fresh air boost the positive effects of exercising (van Cuijck et al. 2013). Beneficial effects of physical exercise are achieved when the heart rate is raised to the point of perspiration, for at least 20 min and three times a week (McDonald and Hodgdon 1991). This was the guideline for this component of the Mindful2Work training, where a day of short physical activity (20 min) is followed by a rest day to promote recovery and vitalize participants, instead of exhausting them even more. During physical exercise, participants are instructed to follow their own pace, in a way that they exert themselves and start to perspire, but not go further than 70 % of their full capacity. Hereby, participants learn to set their own standard, become aware of their limits, and listen to their body.

The yoga component of the Mindful2Work training is based on the yoga style Hatha restorative yoga. This yoga style is a gentle form of yoga which has the objective to bring stress relief and relaxation (Hanson 2011). Hatha restorative yoga is designed for people who need to restore (physically or mentally). This makes it easily accessible and everyone can do it, no matter what their physical limitations are.

The mindfulness meditation component of the Mindful2Work training is based on mindfulness-based stress reduction (MBSR; Kabat-Zinn 1982), mindfulness-based cognitive therapy (MBCT; Segal et al. 2012), and mindfulness: finding peace in a frantic world (Williams and Penman 2011). The mindfulness part of the training consisted of meditation and exercises (experiential learning), inquiry and discussion of the home practices (reflecting), and theory about mindfulness and important themes (psycho-education). In the first three sessions, basic mindfulness skills are cultivated (monitoring, directing and sustaining attention, body and breath awareness). These are a premise for the second part of the training, where mindfulness is build upon these skills. In the second part, a mindful attitude and way of coping with internal and external events is cultivated (dealing with stress and difficulties, self-compassion, and self-care). The mindfulness skills are not only cultivated during the mindfulness part. The participants are invited to carry out the outdoor physical exercises, as well as the yoga, with full attention and awareness while being kind to themselves.

### Measures

#### Feasibility and Acceptability

Intervention and research participation (feasibility) were measured in terms of attendance rates during training sessions and the follow-up session 6 weeks after the training, as well as responses to research measurements. Intervention satisfaction (acceptability) was measured in terms of responses to the evaluation questionnaire administered after the Mindful2Work training.

#### Primary Outcome Measure: Workability

Workability was defined by four characteristics: (1) Total score on the checklist individual strength (CIS), (2) risk for long-term dropout from work (CIS cutoff point), (3) work ability index (WAI), and (4) return to work index (RTWI). The CIS measures different aspects of subjective fatigue and burnout and is validated for the working situation (Beurskens et al. 2000; Vercoulen et al. 1994). The CIS consists of 20 items and is divided over four domains of (work-related) fatigue and exhaustion: subjective fatigue (e.g., ‘I feel tired’), reduced motivation (e.g., ‘I feel no desire to do anything’), reduced activity (e.g., ‘I don’t do much during the day’), and reduced concentration (e.g., ‘My thoughts easily wander’). A cutoff point of ≥76 has been established for employees, who are at increased risk for dropout (long term) from work because of illness (Bültmann et al. 2000). Internal consistency at pre-test in our study was good (*α* = .80 for CIS-total score, *α* = .78 for subjective fatigue, *α* = .78 for reduced motivation, *α* = .78 for reduced activity, and *α* = .90 for reduced concentration). Workability was further assessed by the WAI (Tuomi et al. 1997). Due to the theoretical complexity and practical issues, the single- or double-item question on workability often replaces the WAI in clinical work and research (Ahlstrom et al. 2010). We therefore included two items (‘How do you rate your physical workability at this moment?’ and ‘How do you rate your mental workability at this moment?’). Last, the RTWI was calculated by assessing the ratio of time at work/sick leave (relative to one’s contractual hours) at pre-test, post-test, and both follow-up moments.

#### Secondary Outcome Measures: Anxiety, Depression, Stress, Sleep, and Affect

Symptoms of anxiety and depression were measured by the depression, anxiety, and stress scale (DASS-21; Lovibond and Lovibond 1995). The DASS-21 consists of 21 statements representing three subscales: depression, anxiety, and stress. Example items are ‘I felt that I had nothing to look forward to’ or ‘I felt I was close to panic.’ In addition to the average scores, clinical cutoff points for anxiety disorder and depression, as established in a population of employees absent from work due to mental health problems, for the anxiety subscale (score ≥ 5, sensitivity 92 %) and the depression subscale (score ≥ 12, sensitivity 91 %) were included (Nieuwenhuijsen et al. 2003). Furthermore, the severity labels ‘normal,’ ‘mild,’ ‘moderate,’ ‘severe,’ and ‘extremely severe’ as suggested by Lovibond and Lovibond (1995) were also registered. Internal consistency at pre-test was .67 for the DASS-21 anxiety subscale and .93 for the DASS-21 depression subscale.

General every day experience of stress was measured by the perceived stress scale (PSS; Cohen et al. 1983). The ten-item version of the PSS was used in this study (i.e., ‘I felt nervous and stressed’). Internal consistency at pre-test was .76. Furthermore, stress was also assessed by the stress subscale of the DASS-21 (i.e., ‘I found it hard to wind down’). Internal consistency at pre-test was .82. Last, somatic components of stress were measured by the somatization subscale of the Four-Dimensional Symptom Questionnaire (4DSQ; Terluin 1996). The 4-DSQ consists of 50 items divided over four subscales (distress, depression, anxiety, and somatization). In the current study, only the subscale somatization (16 items) is included since the other domains are covered by other questionnaires. Somatization refers to physical complaints of stress (i.e., ‘Feelings of back pain, neck pain’). Internal consistency of this subscale at pre-test was good, *α* = .74.

The Pittsburgh sleep quality index (PSQI; Buysse et al. 1989) was used to measure subjective perception of sleep quality. The PSQI consists of 19 items, addressing seven components of sleep: sleep quality, sleep latency, sleep duration, habitual sleep efficiency, sleep disturbances, use of sleeping medication, and daytime dysfunction. For the current study, only the sleep quality component (‘How would you rate your overall sleep quality over the past two weeks?’) was included since it was hypothesized that the Mindful2Work training would have a positive effect on one’s subjective experience of sleep quality.

Positive and negative affect were assessed with the positive and negative affect scale (PANAS; Watson et al. 1988). The PANAS consists of 20 words that describe different feelings and emotions (i.e., ‘strong,’ ‘scared,’ ‘inspired,’ ‘active’). Internal consistencies of the positive and negative affect subscales at pre-test were good, *α* = .75 and *α* = .82, respectively.

### Data Analyses

Repeated measures ANOVA’s with post hoc contrasts were carried out to test for the effect of time in both primary and secondary outcome measures. Partial eta squared (*η*_*p*_^2^) was used as a measure of effect size of the overall effect, as well as for effects of scores at post-test, follow-up 6 weeks after training, and follow-up 6 months since the start of the training as compared to scores at pre-test. In accordance with Cohen’s guidelines (1988), *η*_*p*_^2^ = .01 is considered small, *η*_*p*_^2^ = .06 as moderate, and *η*_*p*_^2^ = .14 is considered as a large effect size.

Furthermore, Pearson’s correlations were calculated to assess relationships between intensity of home practice, number of attended sessions, and improvement (changes) in primary outcome measures.

## Results

### Feasibility: Intervention and Research Participation

**Fig. 1:**
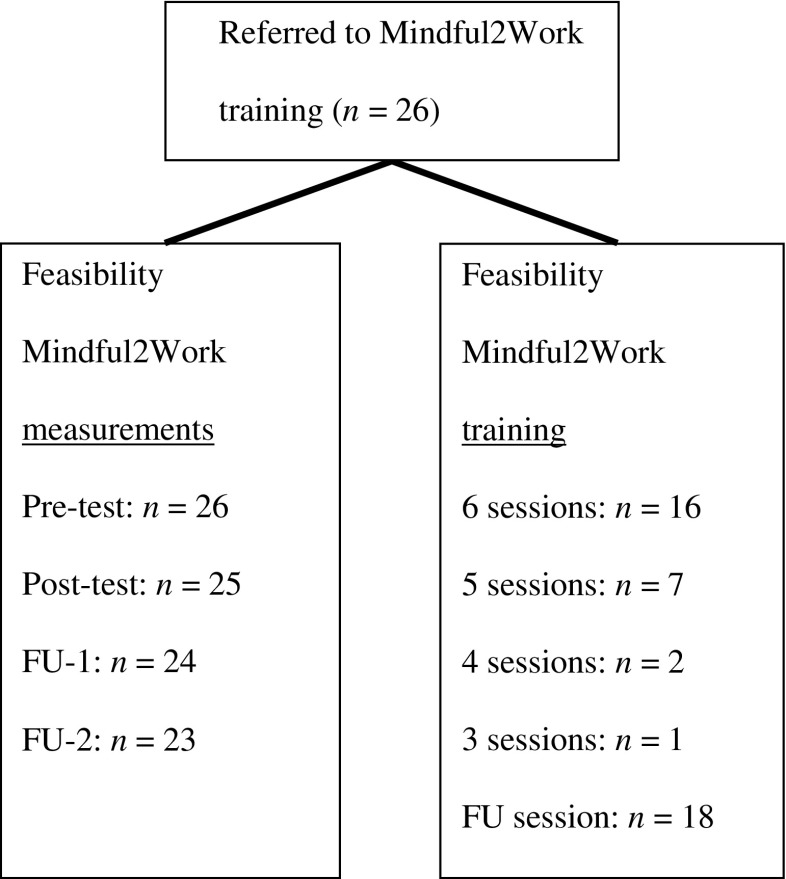
Feasibility of Mindful2Work training adherence and research measurements. *FU-1* follow-up measurement 6 weeks after the Mindful2Work training; *FU-2* follow-up measurement 6 months after the start of the Mindful2Work training

At least five out of the total of six training sessions were followed by 89 % (*n* = 23) of the participants. At the follow-up session 6 weeks later, 69 % (*n* = 18) of the participants were present. This indicates that the Mindful2Work training had a 0 % dropout rate since dropouts were defined as those attending less than four sessions. With respect to the feasibility of the research, one participant was considered a dropout since she only filled in pre-test measurements. All other participants filled in at least pre-test and post-test measurements. For exact feasibility, numbers for training, and research measurements, see Fig. 1.

### Acceptability: Intervention Satisfaction

Ratings about how useful the different sessions and exercises were for the participants are presented in Table 1. Overall, the Mindful2Work training received a grade of 8.1 (scale 1–10, range 7–9), and trainers 8.4 (scale 1–10, range 7–10). Four additional open evaluation questions were administered after the training. *Question 1*. ‘What is your opinion on the Mindful2Work training?’: (very) useful, especially the meditations, eye-opener, very pleasant, relaxing, exactly what I needed, difficult to integrate in daily life due to time it requires, stimulating to carry through some changes in my life, insight that change comes gradual, I started to do more physical exercise (although this was not new to me), information about mindfulness was interesting but brief, I learned how to acquire a deeper state of relaxation and how to deal with stressful situations, could have been longer, met my expectations, the training helps, and I already did yoga but now I pay more attention to stressful places in my body. *Question 2*. ‘What element of the Mindful2Work training did you consider most helpful?’: mindfulness meditations 40.8 %, yoga 22.4 %, physical exercise 14.3 %, combination of the three elements 8.2 %, group process 8.2 %, and psycho-education 6.1 %.*Question 3*. ‘Did you feel the three elements of the Mindful2Work training (first physical exercise, then yoga, and last mindfulness meditations) were a helpful build-up?’: Yes, elements were offered in a good order and balanced combination 95.8 %. One person indicated that mindfulness meditations alone would have been sufficient. *Question 4*. ‘What elements of the Mindful2Work training are you likely to continue?’: Physical exercise + yoga + mindfulness meditations 60 %, physical exercise + mindfulness meditations 12 %, yoga + mindfulness meditations 12 %, physical exercise + yoga 8 %, and mindfulness meditations alone 8 %. Furthermore, directly after training, as well as at both follow-up measurements, participants were asked ‘What, if any, changes do you (still) notice since the Mindful2Work training?’ Answers are summarized in Table 2. According to 92 % of the participants, the changes they reported could be attributed to the Mindful2Work training, and 32 % of those attributed the positive effects to the Mindful2Work training in combination with something else (i.e., other training, less working hours). Furthermore, 32 % (*n* = 8) of the participants reported to have taken part in some other form of training or therapy after the 6-week follow-up session of the Mindful2Work training (physiotherapy *n* = 1, coach for work reintegration *n* = 1, meditation classes *n* = 1, cognitive behavior therapy *n* = 1, schema focused therapy *n* = 1, therapy not further specified *n* = 2, and GP’s assistant’s guidance *n* = 1).

**Table 1:** Evaluation of session themes and exercises of the Mindful2Work training

Theme sessions	Ratings (scale 1–3)
Session 1—from automatic pilot to become aware	*M* = 2.96 (*SD* = 0.20)
Session 2—the body	*M* = 2.79 (*SD* = 0.42)
Session 3—the breath	*M* = 2.84 (*SD* = 0.37)
Session 4—stress!	*M* = 2.84 (*SD* = 0.37)
Session 5—dealing with difficulties	*M* = 2.75 (*SD* = 0.44)
Session 6—taking care of yourself	*M* = 2.73 (*SD* = 0.46)
Exercises	Ratings (scale 1–3)
Sitting meditation	*M* = 2.87 (*SD* = 0.34)
Body scan	*M* = 2.70 (*SD* = 0.47)
Breathing exercise (alternate nostril breathing)	*M* = 2.75 (*SD* = 0.44)
Three-min breathing space	*M* = 2.88 (*SD* = 0.33)
Compassion meditation	*M* = 2.41 (*SD* = 0.59)
Walking meditation	*M* = 2.57 (*SD* = 0.65)
Stress visualization-breathing space practice	*M* = 2.68 (*SD* = 0.48)
Yoga exercises	*M* = 2.72 (*SD* = 0.61)
Boot camp exercises	*M* = 2.73 (*SD* = 0.47)

*1* not so helpful, *2* somewhat helpful, *3* very helpful

**Table 2:** Evaluation: ‘What has changed for you since the Mindful2Work training?’

Post-test	Follow-up 1	Follow-up 2
Sleep better	More focus, clearer choices	I gradually grew stronger
More aware of tense body	Feel more fit	Easier to calm myself
Better able to let go of tense feelings	More aware of tension (physical and mental)	More aware of thoughts and ability to let them go
Know how to cope with stressful situations	Aware of my own well-being and welfare	Awareness of present moment, being in the now
More positive attention for myself	My emotions are better controlled	Yoga and meditation have become part of my life style
I have started working again	Take more time for myself	I have slowed down
Calmer, better able to calm myself when necessary	More sense of control in a hectic environment	Aware of feelings and emotions as passing events
More optimistic, positive	Feeling calmer	Calmer, peace of mind
More aware in general	Better able to calm myself	I am more patient
Create more time for myself	Higher awareness in general	More conscious living
More aware of the present moment	More aware that I need to take care of myself	Acceptance of situation as it currently is
More insight in/closer to myself	Better able to decenter from my emotions	More aware of limits (also at work)
More aware of stressful moments	More aware of negative thoughts	More aware of becoming tense (physical and mental)
More relaxed	More living in the moment	Better able to let go
More self-confidence	More aware of feelings	Taking better care of self
More energy	Less worrying	More aware in daily life
More gentle towards self	I feel stronger	Meditate more often
Less panicky	Better able to let go	More physical exercise
Less somatic complaints	More aware of breathing	Aware of going too fast
Accept instead of hide from thoughts and feelings	More enjoyment of small things	Chose more peaceful moments
More attentive and focused at work	Less painful shoulders, backache	More aware than I previously was
More aware of being judgmental (to self)	I pause or stop more, also at work	More aware of my breathing during worrying
More aware of limits, not always need to give 100 %	Less stressed, better able to deal with stressful moments	Checking in on my own feelings more regularly
More aware of my feelings	Better able to draw limits	Acknowledgment
More aware of relaxed moments	Realization there is more in life than career	Sport and yoga are good for me, mentally and physically
I pause more often	Able to observe negative thoughts/feelings instead of being overwhelmed by them	Very aware of the beneficial effects of physical exercise and meditation
	Acceptance of my flaws	
	Divide energy more evenly	

### Primary Outcome Measure

Averages and standard deviations of all measures at pre-test, post-test, and both follow-up measurements are displayed in Table 3.

**Table 3:** Means and standard deviations of outcome measures at pre-test, post-test, and follow-up measurements of the Mindful2Work training

	Pre-test *M* *(SD)*	Post-test *M* *(SD)*	FU-1 *M* *(SD)*	FU-2 *M* *(SD)*
CIS total	*M =* 97.46; *SD =* 14.50	*M =* 79.21; *SD =* 15.16	*M* = 72.83; *SD* = 18.07	*M* = 66.35; *SD* = 19.48
Fatigue	*M =* 41.79; *SD =* 6.76	*M* = 32.96; *SD* = 9.72	*M* = 30.61; *SD* = 11.26	*M* = 27.17; *SD* = 11.06
Motivation	*M =* 17.58; *SD =* 5.51	*M* = 14.75; *SD* = 4.44	*M* = 13.00; *SD* = 5.44	*M* = 11.43; *SD* = 5.31
Activity	*M =* 14.00; *SD =* 4.34	*M* = 11.67; *SD* = 4.03	*M* = 10.52; *SD* = 3.44	*M* = 9.04; *SD* = 3.50
Concentration	*M =* 24.08; *SD =* 6.86	*M* = 19.83; *SD* = 6.97	*M* = 18.70; *SD* = 6.09	*M* = 18.70; *SD* = 5.60
WAI-physical	*M* = 6.08; *SD* = 1.64	*M* = 6.67; *SD* = 1.83	*M* = 7.17; *SD* = 1.15	*M* = 7.65; *SD* = 1.55
WAI-mental	*M* = 4.88; *SD* = 1.42	*M* = 6.10; *SD* = 1.86	*M* = 6.96; *SD* = 1.33	*M* = 7.04; *SD* = 1.64
DASS total	*M =* 38.58; *SD =* 20.87	*M* = 25.67; *SD* = 11.04	*M* = 20.26; *SD* = 11.65	*n/a*
Depression	*M =* 11.42; *SD =* 9.88	*M* = 7.58; *SD* = 6.27	*M* = 5.48; *SD* = 6.01	*n/a*
Anxiety	*M =* 8.25; *SD =* 5.94	*M* = 6.00; *SD* = 4.76	*M* = 4.17; *SD* = 3.66	*n/a*
Stress	*M =* 18.92; *SD =* 7.71	*M =* 12.08; *SD* = 5.52	*M* = 10.61; *SD* = 5.13	*n/a*
PSS total	*M* = 20.96; *SD* = 5.02	*M* = 16.17; *SD* = 4.78	*M* = 15.22; *SD* = 5.53	*n/a*
4-DSQ somatic	*M =* 12.80; *SD =* 5.58	*M* = 10.76; *SD* = 5.49	*M* = 8.00; *SD* = 4.74	*M* = 8.35; *SD* = 4.05
PSQI sleep quality	*M =* 1.50; *SD =* 0.78	*M =* 1.09; *SD* = 0.61	*M* = 1.43; *SD* = 0.66	*n/a*
PANAS neg. affect	*M =* 26.17; *SD =* 6.45	*M* = 21.67; *SD* = 6.57	*M* = 19.22; *SD* = 5.62	*M* = 18.96; *SD* = 5.15
PANAS pos. affect	*M =* 27.04; *SD =* 5.21	*M* = 31.71; *SD* = 5.08	*M* = 32.30; *SD* = 6.14	*M* = 34.04; *SD* = 6.89

*CIS* checklist individual strength, *DASS* depression anxiety and stress scale, *4DSQ* Four Dimensional Symptoms Questionnaire, *FU-1* follow-up 6 weeks after the Mindful2Work training, *FU-2* follow-up 6 months after the start of the Mindful2Work training, *PANAS* positive and negative affect scale, *PSQI* Pittsburgh sleep quality index, *PSS* perceived stress scale, *WAI* work ability index

#### Workability

Overall, a significant effect of time on work-related fatigue and exhaustion (burnout) was found for CIS total score, *F* (3, 19) = 18.30, *p* < .001, *η*_*p*_^2^ = .74 (very large effect size). Scores on post-test, follow-up 1, and follow-up 2 were significantly lower than those at pre-test, *p* < .001; *η*_*p*_^2^ = .60, *p* < .001; *η*_*p*_^2^ = .69, and *p* < .001; *η*_*p*_^2^ = .71, respectively, meaning that fatigue and exhaustion were largely reduced after the Mindful2Work training and this effect grew even stronger up to 6 months after the start of the training. In line, main effects of time were found for all four CIS subscales: subjective fatigue, *F* (3, 19) = 18.24, *p* < .001, *η*_*p*_^2^ = .74; motivation, *F* (3, 19) = 15.69, *p* < .001, *η*_*p*_^2^ = .71; activity, *F* (3, 19) = 10.24, *p* < .001, *η*_*p*_^2^ = .62; and concentration, *F* (3, 19) = 9.52, *p* < .001, *η*_*p*_^2^ = .60. For subjective fatigue, scores at post-test and both follow-ups were significantly improved as compared to pre-test, *p* < .001; *η*_*p*_^2^ = .59, *p* < .001; *η*_*p*_^2^ = .56, *p* < .001; and *η*_*p*_^2^ = .62, respectively. For energy and motivation (motivation), a similar picture emerged after training. Motivation increased at post-test *p* < .05; *η*_*p*_^2^ = .23, *p* < .001; and further increased at follow-up 1, *η*_*p*_^2^ = .47, and at follow-up 2, *p* < .001; *η*_*p*_^2^ = .67 as compared to pre-test. Also, the feeling of activation, of getting things done (activity) increased at post-test, *p* < .05; *η*_*p*_^2^ = .27, and increased even more at follow-up 1, *p* < .001; *η*_*p*_^2^ = .54, and at follow-up 2, *p* < .001; *η*_*p*_^2^ = .57, as compared to pre-test. And last, the ability to stay focused and concentrate (concentration) significantly improved at post-test, *p* < .001; *η*_*p*_^2^ = .45, follow-up 1, *p* < .001; *η*_*p*_^2^ = .55, and at follow-up 2, *p* < .001; *η*_*p*_^2^ = .47 as compared to pre-test. All effect sizes are very large (*η*_*p*_^2^ > .14 is considered large; Cohen 1988).

Prior to the training, 92 % of the employees (*n* = 22) were at high risk for dropout from work due to illness (CIS total score of ≥76). This was reduced to 67 % (*n* = 16) directly after the training, 44 % (*n* = 10) 6 weeks later and 35 % (*n* = 8) 6 months after the start of the training. Furthermore, significant main effects of time were found for the WAI-physical, *F* (3, 19) = 4.83, *p* < .05, *η*_*p*_^2^ = .43 (very large effect size), with scores on both follow-up measurements being significantly higher than scores on pre-test, *p* < .01; *η*_*p*_^2^ = .33, and *p* < .01; *η*_*p*_^2^ = .39, respectively (*p* = .16; *η*_*p*_^2^ = .09 from pre- to post-test). In line, significant main effects of time were also found for the WAI-mental, *F* (3, 19) = 19.06, *p* < .001, *η*_*p*_^2^ = .75 (very large effect size). Scores at post-test, as well as on both follow-up measurements were significantly improved as compared to pre-test, *p* < .001; *η*_*p*_^2^ = .49, *p* < .001; *η*_*p*_^2^ = .68, and *p* < .001; *η*_*p*_^2^ = .48, respectively. And last, prior to the Mindful2Work training, the participants were working on average 64.7 % of their contract hours per week. After the training, this RTWI increased to 72.8 %; 6 weeks later, this was further increased to 80.5 % and 6 months since the start of the Mindful2Work training, the RTWI was even further increased to 89.0 %. For these long-term follow-up data, one participant could be considered an outlier. She had an unexpected epileptic insult in the follow-up period, for which she was under medical investigation and had to stop working entirely for that time period, whereas directly after the training and 6 weeks later, she was working 100 %. With this participant excluded, the RTWI at follow-up 2 was 93.1 %.

### Secondary Outcome Measures

#### Anxiety and Depression

A main effect of time for anxiety (DASS-21 subscale anxiety) was found, *F* (2, 21) = 4.83, *p* < .05, *η*_*p*_^2^ = .32 (very large effect size). Post hoc comparisons showed a borderline significant decrease of anxiety symptoms at post-test, *p* < .10; *η*_*p*_^2^ = .13, and a significant decrease at follow-up-1, *p* < .01; *η*_*p*_^2^ = .32, as compared to pre-test. Before the Mindful2Work training, 12.5 % of the participants scored in the severe or extremely severe range for anxiety, whereas after the training, this was reduced to 0 %, which was maintained to 6 weeks later. When clinical cutoff points were used to identify people with a very high likelihood of meeting criteria for an anxiety disorder, it was found that before training, 79 % scored on or above this cutoff point, and this was reduced to 54 % after the training. Six weeks later 39 % scored above the clinical cutoff point. Scores on depression (DASS-21 subscale depression) also showed a significant decrease over time, *F* (2, 21) = 7.84, *p* < .01, *η*_*p*_^2^ = .43 (very large effect size). Symptoms of depression were significantly decreased at post-test, *p* < .05; *η*_*p*_^2^ = .23, and at follow-up-1, *p* < .01; *η*_*p*_^2^ = .43, as compared to pre-test. Before the Mindful2Work training, 12.5 % of the participants scored in the severe or extremely severe range for depression, whereas after the training and at 6-week follow-up, this was reduced to 4.2 and 4.3 %, respectively. With respect to clinical cutoff points for depression, at pre-test 33 % scored above this cutoff point, and at post-test and follow-up 1, this was reduced to 17 and 13 %, respectively.

#### Stress

A significant effect for time on PSS total score was found, *F* (2, 21) = 25.04, *p* < .001, and *η*_*p*_^2^ = .71 (very large effect size). Further pairwise comparisons showed that PSS total score was significantly lower at post-test and follow-up 1, as compared to pre-test, with *p* < .001; *η*_*p*_^2^ = .52, and *p* < .001; *η*_*p*_^2^ = .65, respectively. Reductions in stress were confirmed by the other measure of stress, the DASS-21-subscale stress, for which a significant effect of time was found, *F* (2, 21) = 16.10, *p* < .001, and *η*_*p*_^2^ = .61 (very large effect size). Post hoc pairwise comparisons showed that DASS-21-Stress scores were significantly lower at post-test, and further decreased at follow-up 1, as compared to pre-test, with *p* < .01; *η*_*p*_^2^ = .36, and *p* < .001; *η*_*p*_^2^ = .61, respectively. Before the Mindful2Work training, 20.9 % of participants scored in the severe or extremely severe range of stress. After training, this was reduced to 0 % which was maintained at 6-week follow-up. In addition, a main effect of time for somatic stress complaints (4-DSQ somatic) was found, *F* (3, 20) = 8.88, *p* < .01, and *η*_*p*_^2^ = .57 (very large effect size). Post hoc pairwise comparisons further revealed that somatic stress complaints were borderline significantly decreased at post-test, *p* < .10; *η*_*p*_^2^ = .16, and significantly decreased at both follow-up measurements, *p* < .001; *η*_*p*_^2^ = .52, and *p* < .01; *η*_*p*_^2^ = .36, respectively.

#### Sleep

Sleep quality (PSQI) significantly improved over time overall, *F* (2, 19) = 4.48, *p* < .05, and *η*_*p*_^2^ = .32 (very large effect size). Post hoc comparisons showed that sleep was significantly improved at post-test, *p* < .05; *η*_*p*_^2^ = .26, but not at follow-up 1, *p* > .05; *η*_*p*_^2^ = .00.

#### Affect

Positive affect (PANAS-positive) significantly increased over time with a very large effect size, *F* (3, 19) = 13.15, *p* < .001, and *η*_*p*_^2^ = .68. Positive affect significantly increased at post-test, *p* < .001; *η*_*p*_^2^ = .55, at follow-up 1, *p* < .001; *η*_*p*_^2^ = .56, and at follow-up 2, *p* < .001; *η*_*p*_^2^ = .56 as compared to pre-test. In addition, negative affect (PANAS-negative) significantly decreased over time, *F* (3, 19) = 11.14, *p* < .001, and *η*_*p*_^2^ = .64 (very large effect size). Negative affect significantly decreased at post-test, *p* < .001; *η*_*p*_^2^ = .45, at follow-up 1, *p* < .001; *η*_*p*_^2^ = .62, and at follow-up 2, *p* < .001; *η*_*p*_^2^ = .47, as compared to pre-test.

#### Relationship with Intensity of Home Practice and Number of Attended Sessions

During the 6 weeks of training, participants reported to practice a weekly average of 243 min of sport/physical exercise (*SD* = 188), which diminished to 165 min average per week (*SD* = 155) during the first 6 weeks after the training, and 172 min (*SD* = 174) on average per week up to 6 months since the start of training. It is possible that this estimate included outdoor cycling time (to work etc.), since in the Netherlands, particularly in Amsterdam, people’s main form of transport is a bicycle. Yoga was practiced at home for an average of 62 min (*SD* = 36) during the training, 63 min (*SD* = 69) during the first 6 weeks after training, and was reduced to 50 min (*SD* = 59) during the 6-month follow-up period. Mindfulness meditations were practiced at home on average per week for 89 min (*SD* = 63) during the Mindful2Work training, 78 min (*SD* = 89) per week directly after the training, and 73 min (*SD* = 99) per week in the 6-month follow-up period after the training.

However, when prospective correlations between home practice (during the training period and during the first follow-up phase), number of attended sessions, and changes in primary outcome measures (at follow-up 1 and at follow-up 2) were assessed, no significant associations became apparent after application of the Bonferroni-Holmes correction for multiple (24 correlations) testing.

## Discussion

This proof of concept study assessed feasibility and acceptability of the newly developed Mindful2Work training, as well as preliminary effects on workability, anxiety, depression, stress, sleep, and affect in employees suffering from (work-related) stress. In addition, we investigated whether the amount of home practice and number of attended sessions was related to outcome.

We considered attendance and the subjective evaluations to be indicators of feasibility and acceptability. Nearly 90 % of all participants followed five or all six sessions, and dropout rate was zero. It is known from participation in psychological treatment in general that nearly 47 % of clients drop out before the treatment or training is completed (Wierzbicki and Pekarik 1993). We therefore conclude that the Mindful2Work training has a very high feasibility in this sample of participants with burnout related symptoms. Perhaps this high attendance rate was indicative of the level of suffering. Participants were characterized by a high level of burnout related symptoms when they started the Mindful2Work training and were seeking to relieve their suffering. In addition, high conscientiousness and perfection is often seen in people at risk for burnout, which might further explain their consistent participation. Moreover, in most cases, the employer paid for the training costs, which may also have motivated the employees to attend all sessions. Employees were partly on sick leave from work, therefore they perhaps felt the space to attend all sessions, which were mostly held during work time, and for most participants, travel distance was within a range of only 5 km.

The participants gave the Mindful2Work training an average grade of 8.1 out of ten and were highly positive about all three elements of the training and the combination of the three. Participants further clarified many changes that happened in their lives since the Mindful2Work training (i.e., sleep better, more optimistic, more aware of physical tension and therefore better able to let go of it, more insight and understanding of themselves during depression/burnout, better able to cope in stressful situations, more positive attention towards themselves). Most of these changes were still present 6 months after the start of the training. We feel that we can therefore safely conclude that the Mindful2Work training has a very high acceptability.

Since employees that were (self-) referred to this training suffered from complaints that affected their ability to function well at work, the primary outcome measure of this study was workability. Overall, the Mindful2Work training had a very large positive effect on the workability. The risk for long-term dropout from work decreased by nearly 60 %, the mental and physical workability increased, as well as the hours participants returned back to work. Large effects were found immediately after training and lasted, and in most cases, grew even stronger in the long term. Particularly the increase in working hours has obvious financial advantages, since societal costs for people that are absent from work are very high (i.e., Rosch 2001). Inspecting the overall mental and physical workability grade, which went from a low of 4.88 and 6.08, respectively, to a high of 7.04 and 7.65, respectively, also indicated substantial improvements after training.

Treatment as usual for burnout is either person-directed, organization-directed, or a combination of both. Awa et al. (2010) conducted a meta-analysis of all three intervention types and found that 80 % of the included studies led to positive effects on burnout. Duration of interventions ranged from 2 days to 10 months, and effect sizes (only stated in three studies) ranged from small to large. The positive effects of person-directed interventions were maintained in the short term (6 months or less), while a combination with organization-directed interventions had longer lasting effects (12 months and more). The duration of the selected interventions was typically 6 months or less. Interventions that had booster courses (to refresh) had longer lasting effects. However, effects diminished over time in all cases. In comparison to treatment as usual, the effect sizes of the Mindful2Work training were larger and the positive effects of the training were not only maintained but also seemed to extend further in the long term. Furthermore, the duration of the Mindful2Work training was relatively short, compared to treatment as usual, which is favorable not only for regarding cost-effectiveness, but also for accelerating return to work. To compare, a regular MBSR training covers usually around 27 h of training sessions, whereas the Mindful2Work program consisted of 14 h of training sessions.

There are more similar interventions (e.g., the mindful at work programs from Wolever et al. 2012) that also contain physical exercise, yoga, or mindfulness, but comparison to the current study is difficult because the components are usually not combined but studied separately. However, previous positive effects of physical exercise on workability have been shown. For instance, Pohjonen and Ranta (2001) showed that regular physical exercise (9-month training program of twice a week) kept the level of workability index of employees constant after 1 year, while the workability of the control group who did not exercise decreased. During a 5-year period, these changes were maintained for the intervention group, while the workability index of the control group declined three times faster. Furthermore, it was shown that physical activity predicts lower levels of future job burnout, depression, and other mental disorders (Sanchez-Villegas et al. 2008). In line, mindfulness trainings have shown positive effects on workability. Mindfulness decreases the effects of stress in employees (Chaskalson 2011) improves mental well-being (i.e., Brown and Ryan 2003; Carmody and Baer 2008; Chiesa and Serretti 2009), cognitive functioning (Zeidan et al. 2010), and physical health (Davidson et al. 2003; Delgado et al. 2010), which all contribute to mental and physical workability. Research shows that yoga also contributes to this, on a mental (Smith et al. 2007; Wolever et al. 2012) and a physical level (Vera et al. 2009).

Since all three different elements of the training have shown to be effective before, but effect sizes of this combined training appear much higher and longer lasting than what has been reported in the literature with respect to the three separate interventions, one could speculate that this may be due to the synergetic effect of three effective elements. Due to the different elements in the training, stress is targeted on multiple levels. On a physical level, tension is decreased and relaxation and regeneration are promoted. Furthermore, the physical activities are conducted with mindful awareness, and the emphasis lies on a shift from thinking (willpower; “What do I want?”), to feeling (“How am I really doing?”; “What do I need right now?”). The body is a great source of information. By feeling, the connection with the body is restored and the wisdom of the body can be used. Bodily sensations are signals that tell us how we are doing, and also exactly what we need and what our limitations are. Listening to the body and taking care of oneself decreases the tendency to cross or ignore our limits. Besides working with the body, working with the mind is the other level where stress is targeted in the Mindful2Work training. By enhancing attention and less mind wandering to the past or future, more peace of mind and equanimity is established. This was also reflected in participant’s answers to the open evaluation questions. Furthermore, the self-investigation during meditations and exercises provides important insights. Participants learn to take a distance from internal (thoughts, feelings, and physical sensations) and external events and regain freedom in having a choice in how they relate to them. In line, this was emphasized in the evaluations by the participants. Given the fact that body and mind are intertwined and non-stop information exchange takes place, it is likely that working on both levels leads to synergy: the total sum is bigger than the separate parts. This synergy is likely to explain the large effects of this training. This hypothetical synergy is further underlined by the fact that 95 % of the participants considered the three elements a good combination and 60 % wanted to continue with all three of them after the training. Possibly, the mindful exposure to nature during the sport part of the training (boot camp in the park) provides a positive effect in itself which in turn has a continuing positive effect on the physical exercise, yoga, and meditation that follow. Meta-studies of nature-assisted therapies (NAT) confirm these positive effects of exposure to nature for a variety of symptoms and disorders, including stress-related symptoms (Annerstedt and Währborg 2011; Währborg et al. 2014).

In addition to the effects on workability, large immediate, middle long-term, and long-term effects were also found for secondary outcome measures anxiety, depression, stress, sleep problems, and affect. In line with primary outcomes, not only did most effects last up to 6 months after the start of the training, but effects also seemed to ‘grow.’ Participants felt much less anxious, stressed and depressed, suffered from less somatic stress complaints such as shoulder, neck and back aches, slept better, and felt more positive and energetic. Although in the follow-up period after the training and the months after the follow-up session no training sessions were offered, the effects of the training seemed to extend further. It seemed like the seeds of the training were planted and the fruits blossomed even more later on in time. It seems that although the intervention stopped, the tools that were learned in the training were still used and mastery enhanced.

Overall, effects were not related to the amount of home practice or number of sessions attended. In the mindfulness literature, this finding is not uncommon. Although positive associations have been found between intensity of formal home practice (‘prescribed’ home work exercises each week) in MBSR and MBCT courses and outcomes such as rumination and relapse to depression, no relationships were found with amount of informal home practice (any other mindfulness practices, outside of the prescribed home work, i.e., mindful walking the dog, mindful washing the dishes) (Crane et al. 2014; Hawley et al. 2014). The lack of associations in the current study might be explained by the difficulties in measuring home practice. We asked participants to report retrospectively how much they exercised, practiced yoga, and meditation over the past period (instead of keeping a daily diary) and did not differentiate between formal and informal meditation practice. Participants might not have accurately remembered this retrospective time period, and also the term ‘home practice’ might have been somewhat ambiguous. Some participants might have interpreted this as formal meditations only, whereas others might interpret this as covering both formal and informal meditations or not being aware of a difference between the two. In line, it is unclear whether participants distinguished between mindful physical exercise (like in the Mindful2Work training) and general sports. And last, it might be somewhat limited to only look at the practice quantity, whereas brief practices of very high quality (which is difficult to define objectively) might be just as effective, or this might differ per person. Taking all this into account, we feel caution applies when interpreting the lack of correlations between home practice, attended sessions, and changes in outcome measures. A disadvantage of this type of correlational research is that even when amount of practice is related to outcome, due to the inherent bidirectionality of correlational research, it is unclear whether practice leads to good outcome or whether good outcome motivates practice.

Although long-lasting and transformational effects of mindfulness training (Kabat-Zinn; 2003; Singh et al. 2008), yoga (Smith et al. 2007; Vera et al. 2009) and physical exercises (Pohjonen and Ranta 2001) have been shown before, we cannot attribute the long lasting effects solely to the Mindful2work training since around one third of the participants took part in other forms of training after the M2W training. For future studies, these additional treatments should be monitored in greater detail. Also part of the training took place outside; contact with nature and the direct physical sensations of warm and cold, wet and dry, etc. could have perhaps enhanced present moment awareness in itself, and in turn, relieved stress and improved well-being (Annerstedt and Währborg 2011). We also need to be cautious since no control group or wait-list measurement was included, mainly self-reports were used, and the sample size was only small. Although no control group was included, 92 % of the participants reported on the evaluation questionnaire that they attributed the positive effects to the Mindful2Work training, of which 32 % attributed the positive effects to the Mindful2Work training plus another element (i.e., working less, another complementary training). Naturally, some social desirability in participants’ answers should be taken into consideration. From attribution theory, it is known that the locus of causality (whether you attribute success to yourself or to an external agent) significantly influences the outcome (Harvey et al. 2014). Perhaps the low dropout and large effects of the Mindful2Work training can be explained by elements of the attribution theory. Although participants stated that they attributed the success to the training, perhaps this indirectly means they attributed the success to themselves. After all, they were the ones who adhered to all the training sessions and home practices and therefore had legitimate reasons to attribute the success to themselves. This is for instance where mindfulness-based interventions differ from medication treatment. Also important to realize is the severity of symptoms of participants of the current sample. At the start of the Mindful2Work training, 92 % met the criteria for risk for dropout from work, and although this risk was highly reduced 6 months after the training, still around one third of the participants was at risk for dropout. This severity of suffering was further illustrated by the fact that although effects were overall very large, after training, still nearly 40 % scored above the clinical cutoff point for anxiety disorder. These still relatively high rates are also likely to be related to the additional therapies some participants sought after training. For future (more preventative) studies, it would be interesting to see what the effects on stress-related symptoms are in a less severe group, employees that are still fully at work but suffer from stress nevertheless.

For future studies, the inclusion of a (wait-list) control group, a randomized design, and more objective assessments would be of interest. Also, a higher focus on measures of positive aspects would be recommended, such as work satisfaction and work performance. In addition, focus on effects of participants’ own goals is also recommended (i.e., the goal attainment scale) to shed more light on the particular goals the Mindful2Work training is effective for. Lastly, since this proof of concept study was merely a first step, a logical next step besides including a control group could be to examine the mechanisms of change (mediators) of the Mindful2Work training. What are the mechanisms that contributed or mediated these very large effects? A recent systematic review for instance showed that treatment outcome effects of mindfulness training (in that case MBCT) were associated with, predicted by, or mediated by constructs such as mindful awareness, rumination, worry, self-compassion, and affect (Van der Velden et al. 2015).

## References

[CR1] Ahlstrom L, Grimby-Ekman A, Hagberg M, Dellve L (2010). The work ability index and single-item question: associations with sick leave, symptoms, and health—a prospective study of women on long-term sick leave. Scandinavian Journal of Work, Environment & Health.

[CR2] Aikens KA, Astin J, Pelletier KR, Levanovich K, Baase CM, Park YY, Bodnar CM (2014). Mindfulness goes to work: Impact of an online workplace intervention. Journal of Occupational and Environmental Medicine.

[CR3] Annerstedt M, Währborg P (2011). Nature-assisted therapy: systematic review of controlled and observational studies. Scandinavian Journal of Public Health.

[CR4] Awa WL, Plaumann M, Walter U (2010). Burnout prevention: a review of intervention programs. Patient Education and Counseling.

[CR5] Beurskens AJ, Bültmann U, Kant I, Vercoulen JH, Bleijenberg G, Swaen GM (2000). Fatigue among working people: validity of a questionnaire measure. Occupational Environmental Medicine.

[CR6] Brown KW, Ryan RM (2003). The benefits of being present: mindfulness and its role in psychological well-being. Journal of Personality and Social Psychology.

[CR7] Bültmann U, de Vries M, Beurskens AJ, Bleijenberg G, Vercoulen JH, Kant I (2000). Measurement of prolonged fatigue in the working population: determination of a cutoff point for the checklist individual strength. Journal of Occupational Health Psychology.

[CR8] Buysse DJ, Reynolds CF, Monk TH, Berman SR, Kupfer DJ (1989). The Pittsburgh sleep quality index—a new instrument for psychiatric practice and research. Psychiatry Research.

[CR9] Carmody J, Baer RA (2008). Relationships between mindfulness practice and levels of mindfulness, medical and psychological symptoms and well-being in a mindfulness-based stress reduction program. Journal of Behavioral Medicine.

[CR10] Chaskalson M (2011). Positive and negative stress. The mindful workplace: developing resilient individuals and resonant organizations with MBSR.

[CR11] Chiesa A, Serretti A (2009). Mindfulness-based stress reduction for stress management in healthy people: a review and meta-analysis. The Journal of Alternative and Complementary Medicine.

[CR12] Chiesa A, Serretti A (2011). Mindfulness based cognitive therapy for psychiatric disorders: a systematic review and meta-analysis. Psychiatry Research.

[CR13] Cohen J (1988). Statistical power analysis for the behavioral sciences.

[CR14] Cohen S, Kamarck T, Mermelstein R (1983). A global measure of perceived stress. Journal of Health and Social Behavior.

[CR15] Cohen S, Tyrrell DA, Smith AP (1991). Psychological stress and susceptibility to the common cold. New England Journal of Medicine.

[CR16] Conn VS (2010). Anxiety outcomes after physical activity interventions. Nursing Research.

[CR17] Conn VS (2010). Depressive symptom outcomes of physical activity interventions: meta-analysis findings. Annals of Behavioral Medicine.

[CR18] Conn VS, Hafdahl AR, Cooper PS, Brown LM, Lusk SL (2009). Meta-analysis of workplace physical activity interventions. American Journal of Preventive Medicine.

[CR19] Coyl DD, Roggman LA, Newland LA (2002). Stress, maternal depression, and negative mother–infant interactions in relation to infant attachment. Infant Mental Health Journal.

[CR20] Crane C, Crane RS, Eames C, Fennell MJV, Silverton S, Williams JMG (2014). The effects of amount of home meditation practice in mindfulness based cognitive therapy on hazard of relapse to depression in the staying well after depression trial. Behavior Research and Therapy.

[CR21] Davidson RJ, Kabat-Zinn J, Schumacher J, Rosenkranz M, Muller D, Santorelli SF (2003). Alterations in brain and immune function produced by mindfulness meditation. Psychosomatic Medicine.

[CR22] Delgado LC, Guerra P, Perakakis P, Vera MN, del Paso GR, Vila J (2010). Treating chronic worry: psychological and physiological effects of a training programme based on mindfulness. Behaviour Research and Therapy.

[CR23] DiLorenzo TM, Bargman EP, Stucky-Ropp R, Brassington GS, Frensch PA, LaFontaine T (1999). Long-term effects of aerobic exercise on psychological outcomes. Preventive Medicine.

[CR24] European Agency for Safety and health at Work (2014). OSH in figures: stress at work—facts and figures.

[CR25] Fjorback LO, Arendt M, Ornbol E, Fink P, Walach H (2011). Mindfulness-based stress reduction and mindfulness-based cognitive therapy—a systematic review of randomized controlled trials. Acta Psychiatrica Scandinavica.

[CR26] Formsma AR, de Bruin EI, Bögels SM (2015). Mindful2Work training protocol: a combination of mindfulness meditation, yoga, and physical exercise. Internal publication.

[CR27] Granath J, Ingvarsson S, von Thiele U, Lundberg U (2006). Stress management: a randomized study of cognitive behavioural therapy and yoga. Cognitive Behaviour Therapy.

[CR28] Gura ST (2002). Yoga for stress reduction and injury prevention at work. Work: A Journal of Prevention, Asessment and Rehabilitation.

[CR29] Hammen C (2004). Stress and depression. Annual Review of Clinical Psychology.

[CR30] Hanson J (2011). Relax and renew: restful yoga for stressful times.

[CR31] Harvey P, Madison K, Martinko M, Crook TR, Crook TA (2014). Attribution theory in organizational sciences: the road traveled and the path ahead. The Academy of Management Perspectives.

[CR32] Hassmén P, Koivula N, Uutela A (2000). Physical exercise and psychological well-being: a population study in Finland. Preventive Medicine.

[CR33] Hawley LL, Schwartz D, Bieling PJ, Irving J, Corcoran K, Farb NAS (2014). Mindfulness practice, rumination and clinical outcome in mindfulness-based treatment. Cognitive Therapy and Research.

[CR34] Henriques G, Keffer S, Abrahamson C, Horst SJ (2011). Exploring the effectiveness of a computer-based heart rate variability biofeedback program in reducing anxiety in college students. Applied Psychophysiology Biofeedback.

[CR35] Hofmann SG, Sawyer AT, Witt AA, Oh D (2010). The effect of mindfulness-based therapy on anxiety and depression: a meta-analytic review. Journal of Consulting and Clinical Psychology.

[CR36] Kabat-Zinn J (1982). An out-patient program in behavioral medicine for chronic pain patients based on the practice of mindfulness meditation: theoretical considerations and preliminary results. General Hospital Psychiatry.

[CR37] Kabat-Zinn J (2003). Mindfulness-based interventions in context: past, present, and future. Clinical Psychology: Science and Practice.

[CR38] Kabat-Zinn J, Lipworth L, Burney R (1985). The clinical use of mindfulness meditation for the self-regulation of chronic pain. Journal of Behavioral Medicine.

[CR39] Kalia M (2002). Assessing the economic impact of stress-the modern day hidden epidemic. Metabolism, Clinical and Experimental.

[CR40] Khalsa SBS (2004). Treatment of chronic insomnia with yoga: a preliminary study with sleep-wake diaries. Applied Psychophysiology Biofeedback.

[CR41] LaPerriere AR, Antoni MH, Schneiderman N, Ironson G, Klimas N, Caralis P (1990). Exercise intervention attenuates emotional distress and natural killer cell decrements following notification of positive serologic status for HIV-1. Biofeedback and Self-Regulation.

[CR42] Leone SS, Wessely S, Huibers MJH, Knottnerus JA, Kant I (2011). Two sides of the same coin? On the history and phenomenology of chronic fatigue and burnout. Psychology & Health.

[CR43] Li AW, Goldsmith CAW (2012). The effects of yoga on anxiety and stress. Alternative Medicine Review.

[CR44] Lovibond SH, Lovibond PF (1995). Manual for the depression anxiety stress scale.

[CR45] McDonald DG, Hodgdon JA (1991). The psychological effects of aerobic fitness training.

[CR46] McEwen BS, Sapolsky RM (1995). Stress and cognitive function. Current Opinion in Neurobiology.

[CR47] Mino Y, Babazono A, Tsuda T, Yasuda N (2006). Can stress management at the workplace prevent depression? A randomized controlled trial. Psychotherapy and Psychosomatics.

[CR48] Mothes H, Klaperski S, Seelig H, Schmidt S, Fuchs R (2014). Regular aerobic exercise increases dispositional mindfulness in men: a randomized controlled trial. Mental Health and Physical Activity.

[CR49] Netterstrøm B, Conrad N, Bech P, Fink P, Olsen O, Rugulies R, Stansfeld S (2008). The relation between work-related psychosocial factors and the development of depression. Epidemiologic Reviews.

[CR50] Nieuwenhuijsen K, de Boer AGEM, Verbeek JHAM, Blonk RWB, van Dijk FJH (2003). The depression anxiety stress scales (DASS): detecting anxiety disorder and depression in employees absent from work because of mental health problems. Occupational Environmental Medicine.

[CR51] Pober DM, Braun B, Freedson PS (2004). Effects of a single bout of exercise on resting heart rate variability. Medicine and Science in Sports and Exercise.

[CR52] Pohjonen T, Ranta R (2001). Effects of worksite physical exercise intervention on physical fitness, perceived health status, and work ability among home care workers: five-year follow-up. Preventive Medicine.

[CR53] Raub JA (2002). Psychophysiologic effects of Hatha yoga on musculoskeletal and cardiopulmonary function: a literature review. Journal of Alternative and Complementary Medicine.

[CR54] Reed J, Buck S (2009). The effect of regular aerobic exercise on positive-activated affect: a meta-analysis. Psychology of Sport and Exercise.

[CR55] Richardson KM, Rothstein R (2008). Effects of occupational stress management intervention programs: a meta-analysis. Journal of Occupational Health Psychology.

[CR56] Rosch PJ (2001). The quandary of job stress compensation. Health and Stress.

[CR57] Ross A, Thomas S (2010). The health benefits of yoga and exercise: a review of comparison studies. Journal of Alternative and Complementary Medicine.

[CR58] Sadeh A, Keinan G, Daon K (2004). Effects of stress on sleep: the moderating role of coping style. Health Psychology: Official Journal of the Division of Health Psychology.

[CR59] Sanchez-Villegas A, Ara I, Guillen-Grima F, Bes-Rastrollo M, Varo-Cenarruzabeitia JJ, Martinez-Gonzalez MA (2008). Physical activity, sedentary index, and mental disorders in the SUN cohort study. Medicine and Science in Sports and Exercise.

[CR60] Schneiderman N, Ironson G, Siegel SD (2005). Stress and health: psychological, behavioral, and biological determinants. Annual Review of Clinical Psychology.

[CR61] Segal ZV, Williams JM, Teasdale JD (2012). Mindfulness based cognitive therapy for depression: a new approach to preventing relapse.

[CR62] Shanafelt TD, Hasan O, Dyrbye LN, Sinsky C, Satele D, Sloan J (2015). Changes in burnout and satisfaction with work-life balance in physicians and the general US working population between 2011 and 2014. Mayo Clinic Proceedings.

[CR63] Siegel RD, Germer CK, Olendzki A (2009). Clinical handbook of mindfulness.

[CR64] Singh NN, Lancioni GE, Wahler RG, Winton AS, Singh J (2008). Mindfulness approaches in cognitive behavior therapy. Behavioural and Cognitive Psychotherapy.

[CR65] Smith C, Hancock H, Blake-Mortimer J, Eckert K (2007). A randomised comparative trial of yoga and relaxation to reduce stress and anxiety. Complementary Therapies in Medicine.

[CR66] Stansfeld S, Candy B (2006). Psychosocial work environment and mental health—a meta-analytic review. Scandinavian Journal of Work, Environment & Health.

[CR67] Terluin B (1996). De Vierdimensionale Klachtenlijst (4DKL). Een vragenlijst voor het meten van distress, depressie, angst en somatisatie [The Four-Dimensional Symptom Questionnaire (4DSQ). A questionnaire to measure distress, depression, anxiety, and somatization]. Huisarts & Wetenschap.

[CR68] Tuomi K, Ilmarinen J, Jahkola A, Katajarinne L, Tulkki A (1997). Work ability index. Institute of Occupational Health.

[CR69] van Cuijck J, Holterman A, Hettinga F (2013). Bootcamp. Fitnessen in de buitenlucht [Bootcamp. Fitness outdoors in fresh air]. Sportgericht.

[CR70] Van den Heuvel SG, Boshuizen HC, Hildebrandt VH, Blatter BM, Ariëns GAM, Bongers PM (2003). Sporten, type werk, arbeidsverzuim en welbevinden: resultaten van een 3-jarige follow-up studie. Center for Epidemiological Studies Depression.

[CR71] Van der Velden AM, Kuyken W, Wattar U, Crane C, Pallesen KJ, Dahlgaard J (2015). A systematic review of mechanisms of change in mindfulness-based cognitive therapy in the treatment of recurrent major depressive disorder. Clinical Psychology Review.

[CR72] Vera FM, Manzaneque JM, Maldonado EF, Carranque GA, Rodriguez FM, Blanca MJ (2009). Subjective sleep quality and hormonal modulation in long-term yoga practitioners. Biological Psychology.

[CR73] Vercoulen JH, Swanink C, Fennis JF, Galama J, van der Meer JW, Bleijenberg G (1994). Dimensional assessment of chronic fatigue syndrome. Journal of Psychosomatic Research.

[CR74] Währborg P, Petersson IF, Grahn P (2014). Nature-assisted rehabilitation for reactions to severe stress and/or depression in a rehabilitation garden: long-term follow-up including comparisons with a matched population-based reference cohort. Journal of Rehabilitation Medicine.

[CR75] Watson D, Clark LA, Tellegen A (1988). Development and validation of brief measures of positive and negative affect: the PANAS scales. Journal of Personality and Social Psychology.

[CR76] Wessely S, Sharpe M, Hotopf M (1998). Chronic fatigue and its syndromes.

[CR77] West J, Otte C, Geher K, Johnson J, Mohr DC (2004). Effects of Hatha yoga and African dance on perceived stress, affect, and salivary cortisol. Annals of Behavioral Medicine: A Publication of the Society of Behavioral Medicine.

[CR78] Wierzbicki M, Pekarik G (1993). A meta-analysis of psychotherapy dropouts. Professional Psychology: Research and Practice.

[CR79] Williams M, Penman D (2011). Mindfulness: a practical guide to finding peace in a frantic world.

[CR80] Wolever RQ, Bobinet KJ, McCabe K, Mackenzie ER, Fekete E, Kusnick CA (2012). Effective and viable mind-body stress reduction in the workplace: a randomized controlled trial. Journal of Occupational Health Psychology.

[CR81] Youngstedt SD, O’Connor PJ, Dishman RK (1997). The effects of acute exercise on sleep: a quantitative synthesis. Sleep: Journal of Sleep Research & Sleep Medicine.

[CR82] Zeidan F, Johnson SK, Diamond BJ, David Z, Goolkasian P (2010). Mindfulness meditation improves cognition: evidence of brief mental training. Consciousness and Cognition.

